# Robust interlaboratory reproducibility of a gene expression signature measurement consistent with the needs of a new generation of diagnostic tools

**DOI:** 10.1186/1471-2164-8-148

**Published:** 2007-06-07

**Authors:** Robert A Ach, Arno Floore, Bo Curry, Vladimir Lazar, Annuska M Glas, Rob Pover, Anya Tsalenko, Hugues Ripoche, Fatima Cardoso, Mahasti Saghatchian d'Assignies, Laurakay Bruhn, Laura J Van't Veer

**Affiliations:** 1 Molecular Technology Lab, Agilent Laboratories, Agilent Technologies, 5301 Stevens Creek Blvd., Santa Clara, CA 95051, USA; 2 Agendia BV, Slotervaart Medical Center 9D, Louwesweg 6, 1066 EC Amsterdam, The Netherlands; 3 Institut Gustave-Roussy, 39 rue Camille Desmoulins, 94805 Villejuif Cedex, France; 4 Institut Jules Bordet, 121 Blvd de Waterloo, B-1000 Brussels, Belgium

## Abstract

**Background:**

The increasing use of DNA microarrays in biomedical research, toxicogenomics, pharmaceutical development, and diagnostics has focused attention on the reproducibility and reliability of microarray measurements. While the reproducibility of microarray gene expression measurements has been the subject of several recent reports, there is still a need for systematic investigation into what factors most contribute to variability of measured expression levels observed among different laboratories and different experimenters.

**Results:**

We report the results of an interlaboratory comparison of gene expression array measurements on the same microarray platform, in which the RNA amplification and labeling, hybridization and wash, and slide scanning were each individually varied. Identical input RNA was used for all experiments. While some sources of variation have measurable influence on individual microarray signals, they showed very low influence on sample-to-reference ratios based on averaged triplicate measurements in the two-color experiments. RNA labeling was the largest contributor to interlaboratory variation.

**Conclusion:**

Despite this variation, measurement of one particular breast cancer gene expression signature in three different laboratories was found to be highly robust, showing a high intralaboratory and interlaboratory reproducibility when using strictly controlled standard operating procedures.

## Background

Gene expression analysis with DNA microarrays has been used to develop molecular taxonomies of various types of cancers [1-16]. Small gene sets or signatures of tens to hundreds of genes have been examined for their potential utility in defining tumor subtypes and providing specific prognostic or diagnostic information. One factor that will influence the capability to fully realize the potential utility of these signatures for biomedical research, toxicogenomics, pharmaceutical development, and diagnostics is the reproducibility of the technology used to measure them.

Several studies have examined the reproducibility of gene expression analysis by DNA microarrays across different laboratories. One study used aliquots of a common mouse liver and pooled RNA, and compared gene expression measurements made in seven laboratories using a total of 12 microarray platforms [17]. They found that correlations were highest between labs when the same platform was used with standardized protocols. A second study measured gene expression in a set of four knockout human cell lines across ten laboratories using three different microarray platforms [18]. They found that the particular laboratory which performed the analysis had a greater effect on the precision than did the choice of platform, and the results from the best-performing labs agreed fairly well. A third study done in four laboratories using the same platform to analyze tumor tissue blocks, cell lines, and RNA samples found that correlation within laboratories was only slightly better than correlation between laboratories, with correlations weakest for genes expressed at low levels [19]. More recently, the Microarray Quality Control project (MAQC) compared gene expression measurements of two RNA samples using a number of microarray platforms, as well as alternative technologies, and demonstrated intraplatform consistency and interplatform concordance in terms of genes differentially expressed [20]. A related study found consistency among microarray platforms at different sites using 36 different RNAs from rats treated with three chemicals [21]. Neither of these two recent studies examined whether the variation seen between laboratories was due to the labeling or hybridization steps, or both. While these papers give a general overview of the reproducibility of microarray-based gene expression profiling across a variety of platforms, they focused on the overall reproducibility of measurements made with arrays containing probes designed to measure the majority of known human transcripts, rather than on the reproducibility of gene expression signatures composed of relatively small numbers of genes analyzed on a smaller, targeted array.

In this study, we examined the interlaboratory reproducibility of a specific 70-gene breast cancer signature [1,2], recently developed into a diagnostic tool (MammaPrint^®^, Agendia BV) [14], using the same microarray platform and standardized protocols for labeling and hybridization across three different laboratories. In particular, we examined the level and primary sources of variability between technical replicates using a small array containing probes that measure only a fraction of known human transcripts. In order to better understand the degree and sources of errors attributable to the measurement itself, independent of any biological variability among the samples, we assayed aliquots of the same four breast tumor RNAs in all three laboratories. We specifically measured the variability introduced by each step of the microarray analysis protocols: labeling, hybridization, and scanning. We found that the sample labeling was the largest source of technical variability in this study. However, this variability did not have any significant influence on the overall 70-gene breast cancer signature correlation values, which were quite robust within and between laboratories.

## Results

### Experimental setup

**Figure 1:**
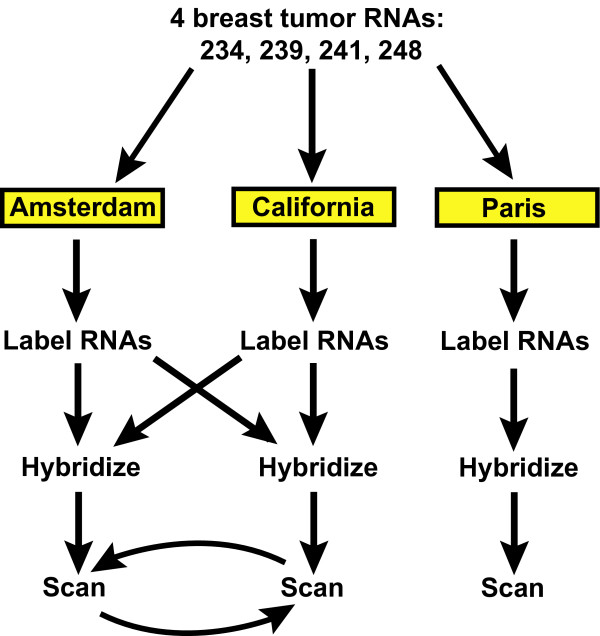
**Outline of experimental design**. All four tumor RNAs plus the reference RNA were amplified and labeled twice with each dye, in both the Amsterdam and California laboratories. Half of the labeled material was exchanged between the two labs, and samples labeled locally and in the other laboratory were hybridized in replicate, and scanned. Slides were shipped to the other laboratory for rescanning. In the third lab (Paris), the tumor samples were independently labeled and hybridized three times.

To compare DNA microarray data reproducibility within and between laboratories, we employed the experimental scheme shown in Figure 1. Aliquots of the same preparations of total RNA from four different human breast tumors were given to three laboratories, one in California, one in Amsterdam, and one in Paris.

In the first phase of this study, we examined the reproducibility of microarray measurements between the California and Amsterdam laboratories. Both laboratories amplified and labeled each RNA sample, and sent aliquots of each labeled product to the other laboratory. Both laboratories then hybridized all the samples labeled in both labs, scanned the slides, and then shipped the slides to the other laboratory for rescanning. The same lot of labeling kits and microarrays were used in both labs. In this manner we could compare the intra- and inter-laboratory variations in each step of the microarray assay, starting with purified total RNA.

Each slide contained eight individual microarrays, which could be hybridized separately. The hybridization setup is shown in Table 1. Both labs hybridized each tumor RNA labeled in each lab in dye flip pairs against the reference. Each site hybridized replicates of the two separate slides on two different days, for a total of four slides per lab.

**Table 1:** Hybridization slide setup. Setup of slides hybridized in Amsterdam and California.

**Slide Number**	**Array Number**	**Cy3 Sample**	**Cy5 Sample**	**Labeling**
1	1	Reference	234	California
1	2	234	Reference	California
1	3	Reference	234	Amsterdam
1	4	234	Reference	Amsterdam
1	5	Reference	239	California
1	6	239	Reference	California
1	7	Reference	239	Amsterdam
1	8	239	Reference	Amsterdam
2	1	Reference	241	California
2	2	241	Reference	California
2	3	Reference	241	Amsterdam
2	4	241	Reference	Amsterdam
2	5	Reference	248	California
2	6	248	Reference	California
2	7	Reference	248	Amsterdam
2	8	248	Reference	Amsterdam

### Signals correlate extremely well between replicate hybridizations

**Figure 2:**
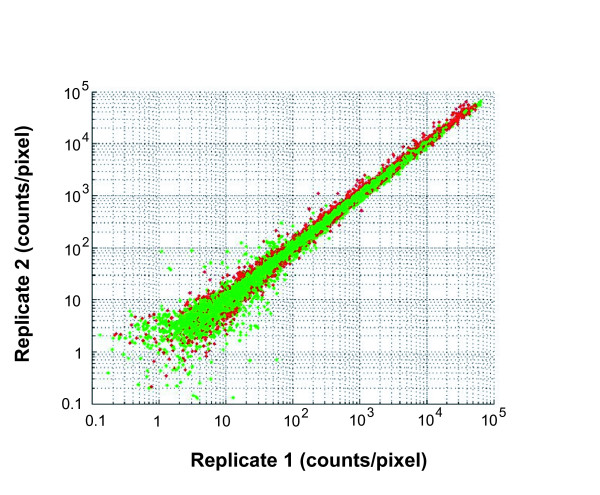
**Replicate correlations for tumor 248**. Plot shows signals from all background subtracted non-control features of 8 replicate hybridization pairs (16 arrays total) for tumor 248. All of the individual features from all of the16 arrays are plotted. One of each replicate pair is plotted on the x-axis, the other is on the y-axis. Green data points are the Cy3 channel, red data points are the Cy5 channel.

Variability among microarray assays might arise from differences between labeled samples, between the arrays themselves, or between hybridization conditions. A replicate hybridization is defined here as a pair of assays for which the sample labeling and hybridization conditions are held constant; that is, aliquots from a single labeling reaction are hybridized to different arrays at the same location. Comparison of replicate hybridizations allows us to determine the noise attributable to hybridization, washing, and scanning, and to variations among the arrays themselves. Contributions to noise include an additive background, a proportional precision, and a stochastic element. In Figure 2 we compare the background-subtracted green (Cy3) and red (Cy5) signals for each of the eight pairs of hybridization replicates of tumor 248. All three sources of noise are evident in the plot: a consistent proportional noise of a few percent, increasing as the signals approach the background noise level (2–5 counts), and a smattering of single-feature outliers. The Pearson correlation reflects all these noise sources, while remaining insensitive to normalization issues. For tumor 248, seven out of eight of the replicate pairs showed Pearson correlation values of > 0.993 in both signal and reference channels, while the other replicate pair had a correlation of 0.983. For the other three tumors, all samples had replicate correlations greater than 0.988, with all but two replicates above 0.993 (Additional file 1). These results indicate that the signals from replicate hybridizations correlated extremely well for genes expressed at all intensity levels measured.

### Scanners correlate extremely well between sites

**Figure 3:**
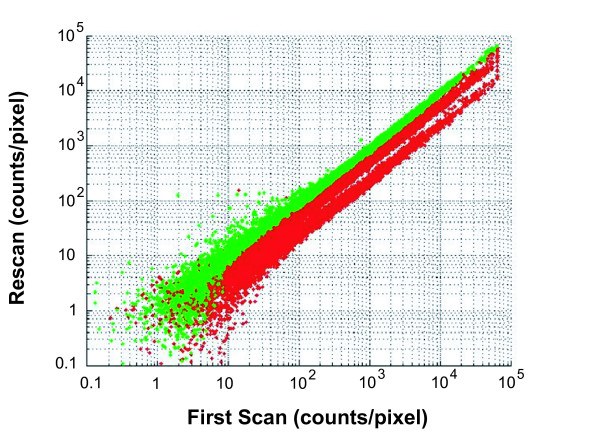
**Scan/rescan correlations for tumor 248**. Plot shows background subtracted signals from the original laboratory scan (x-axis) plotted against the signals from the rescan performed in the other laboratory. All of the individual features from all of the16 arrays are plotted. All 16 arrays for tumor 248 were scanned in the hybridization lab, then shipped to the other lab and rescanned (32 scans total from 16 arrays, on 2 slides). Green data points are from the Cy3 channel, red data points are from the Cy5 channel.

In order to determine whether differences between microarray scanners introduce significant variability into the results, we scanned the hybridized arrays at each site and then shipped them to the other site for rescanning. Figure 3 compares the scan and the rescan for the tumor 248 hybridizations. The signals from the original scan of each of the 16 arrays are plotted against the rescans in green (Cy3) and red (Cy5). The Cy3 signals correlated extremely well between the scan and rescan, regardless of whether the slide was first scanned in Amsterdam or California (Pearson correlation >0.995, slope = 0.97). The Cy5 signals correlated less well, and the signals were always much lower on the rescanned slide, especially for slides scanned first in Amsterdam. This was likely due to degradation of the Cy5 during shipment of the slide between labs, possibly caused by atmospheric ozone [22]. Hybridized slides for the other tumor RNAs showed similar results (data not shown). We conclude that the scanner adds virtually no variability to the array results, as the variability seen in the Cy5 channel is due to shipment of hybridized slides, which typically does not occur in a standard microarray experiment.

### 70-gene signature values correlate between different hybridizations

As a biologically relevant way of measuring the reproducibility of the microarray results, we computed the 70-gene breast cancer signature correlation value as previously described [14] for each dye-swapped pair of arrays. The 70-gene signature correlation value is determined by taking the weighted average of the log_10 _ratios for each of the triplicate probes for each of the 70 genes, and then determining the cosine correlation of the average log_10 _ratios for the 70 genes in the particular tumor sample with the average profile of these genes in tumors from a specific, defined set of patients. This procedure eliminates the effect of random variation in microarray signal strength between probe replicates [14]. The variability of this signature correlation value among the tumor hybridization dye swap pairs under different conditions is a good measure of overall variability in the microarray assay.

**Figure 4:**
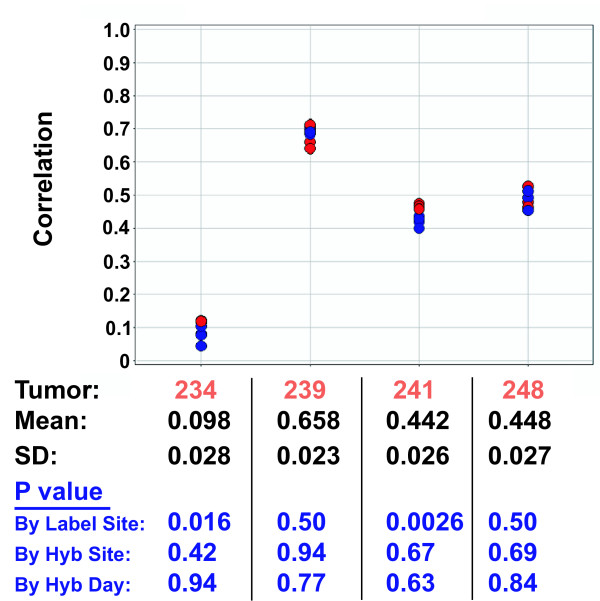
**70 gene signature correlation values**. The 70-gene signature correlation values for the four tumors were determined for each hybridization; these values indicate the correlation of the log ratios of the 70 signature genes from the tumor sample with the average log ratios from a previously defined set of patients [14]. The correlation values for each dye-swapped pair (y-axis) are plotted for each of the four tumors (x-axis). Red data points were labeled in Amsterdam, while blue data points were labeled in California. The mean and standard deviations of the correlation values for each tumor are indicated beneath the plot. Each set of hybridizations for each tumor was divided into two groups, based either on hybridization site, labeling site, or hybridization day. An ANOVA was then performed on the 70 gene signature correlation values obtained in the hybs for both groups, and the resulting P values for each tumor are shown.

Figure 4 shows the eight signature correlation values for each of the eight dye swap pairs of hybridizations of each of the four tumors. The correlation values for each tumor clustered quite tightly, indicating only a small amount of variation in the assay. Even tumor 248, which had the replicate pair with the lowest Pearson correlation (0.983), shows tight clustering of the results from all replicates, indicating the slightly lower Pearson correlation of the one replicate pair does not influence the 70-gene signature correlation value. The results in Figure 4 are colored by labeling site, and the correlation values for tumors 234 and 241 seem to show some systematic variation in the results, with correlation values from samples labeled in Amsterdam being higher than those labeled in California. In order to determine whether there is any statistically significant bias in the correlation values depending on the labeling or hybridization site, we classified the dye-swap pairs for each tumor, according to hybridization site, labeling site, and hybridization day. We then performed an ANOVA analysis to determine whether any of these classes differ significantly in their correlation value means, as reflected in the ANOVA P values. We found that there were no significant differences between the values obtained at different hybridization sites, or on different hybridization days (regardless of site), indicating that the site or day of hybridization did not contribute any systematic variability to the assay. However, tumors 234 and 241 showed a small but statistically significant difference (P value < 0.05) between labeling sites.

### Small differences are seen due to labeling site

**Figure 5:**
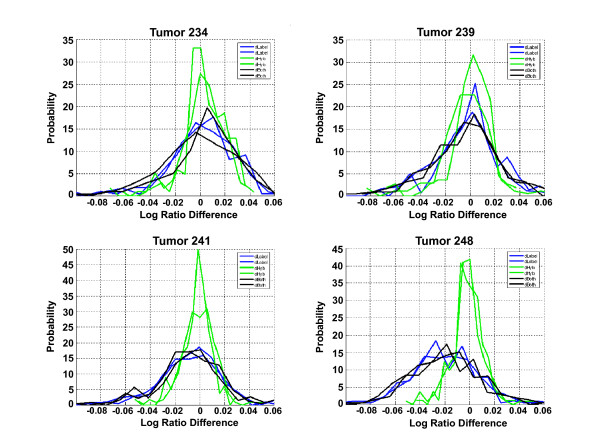
**Distribution of log_10 _ratio differences between conditions for all four tumors**. Distributions of log_10 _ratio differences for the 182 of the 232 genes that had signals significantly above background (signals > 15) are plotted. Each set of log_10 _ratios were compared with another set by subtracting the log_10 _ratios of one set from those of the other to get a set of 182 log_10 _ratio differences. The green distributions compare arrays with the same labeled sample, hybridized in different laboratories. The blue distributions compare arrays labeled at different locations but hybridized at the same location. The black distributions compare arrays with different labeled samples, hybridized in different locations. Each curve is a probability distribution (normalized histogram) of the differences between the average log_10 _ratios of the 182 probes in one condition, and their average in the other condition.

In order to further examine the differences between labeling sites, we averaged the log_10 _ratios of signature probes from the four arrays (two dye-swap pairs) that shared the same labeling and hybridization location, as there is little systematic variation between replicate hybridizations (Figure 2). This resulted in four sets of averaged, dye-bias corrected log_10 _ratios, corresponding to the four combinations of labeling and hybridization locations: Amsterdam labeled/hybridized, California labeled/hybridized, Amsterdam labeled/California hybridized, and California labeled/Amsterdam hybridized. Averaging dye-swapped pairs in this manner eliminates systematic dye bias and reduces random variation, allowing the small differences between samples labeled at the two sites to be observed. These small differences between log_10 _ratios of the samples can be clearly seen by examining the differences between the averaged log_10 _ratios of probes between two different combinations of labeling/hybridization sites. Figure 5 shows plots of the distributions of such log_10 _ratio differences for the 182 of the 232 probes on the array corresponding to the breast cancer associated genes [1] that had signals significantly above background. Each of the curves in Figure 5 is the probability distribution (normalized histogram) of the differences between the average log_10 _ratios of the significant probes in one condition, and their average in the other condition. The green distributions compare the arrays with the same labeled sample, but hybridized in different laboratories. These distributions are very narrow, and are centered around zero, indicating there is no systematic difference depending on the hybridization site. The blue distributions compare arrays labeled at different locations, but hybridized in the same laboratory, and the black distributions were with different labeled material, hybridized in different laboratories. These distributions are wider, indicating the log_10 _ratios show more variance, and are also not always centered at zero, indicating a systematic difference depending on the labeling reaction, but not on the hybridization site. Clearly it mattered little where the arrays were hybridized, but the log_10 _ratios differ depending on where they were labeled. These differences were still relatively small however, as a log_10 _ratio difference of 0.02 is equivalent to a 5% difference in the actual expression ratio.

**Figure 6:**
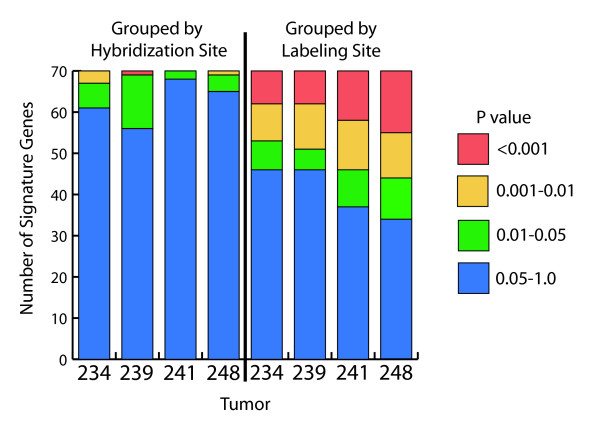
**P values from ANOVA analysis of each of the 70 signature genes**. For each tumor the log_10 _ratios of the 70 signature genes were averaged for each dye-swapped hybridization pair, after reversing the sign of one of the dye swaps. An ANOVA analysis was then performed for each individual gene for each tumor, to determine if the log ratios for each gene varied by hybridization site or by labeling site. The plots show the number of genes for each tumor having P values of < 0.001, 0.001–0.01, 0.01–0.05, and 0.05–1.0 from the ANOVA analysis, when grouped by hybridization site (left) or by labeling site (right).

We next asked whether the residual variation in the correlation values between labeling sites (Figure 4) is distributed across all the signature genes, or is due to a particular subset of genes that consistently vary between labeling sites. To investigate this, we performed an ANOVA analysis on the log_10 _ratios for each of the 70 signature genes separately, to see if they varied significantly between hybridization or labeling sites. A synopsis of the ANOVA P values determined for each tumor is shown in Figure 6. When the hybridizations were grouped by hybridization site, the number of genes showing a statistically significant difference between the two sites (P value < 0.05) ranged from 2 (tumor 241) to 14 (tumor 239). Four of the 70 genes in each signature would be expected to exhibit P values of < 0.05 by chance alone (i.e. 0.05*70). In contrast, when the hybridizations were grouped by labeling site, the number of genes showing a statistically significant difference was much higher, ranging from 24 (tumors 234 and 239) to 36 (tumor 248). Thus many of the 70 signature genes in all four tumors showed significant differences between labeling sites, even though the signature correlation values only showed significant differences between labeling sites for tumors 234 and 241. Further analysis showed that 60 out of the 70 genes varied in at least one tumor, and only five were significantly different in all four tumors. This suggests the variation in labeling was due to increased noise, rather than some sort of systematic variation.

### 70-gene signature values correlate using different reagent lots at a third laboratory

The assays performed in the California and Amsterdam sites used the same batch of arrays, dye-NTPs, and labeling kits in order to minimize differences between the two laboratories. To further look at the variability of the system, we analyzed the same four tumor RNAs in a third laboratory (located in Paris), at a time several months after the initial comparison was finished, using a different lot of microarrays and different lots of labeling reagents.

**Figure 7:**
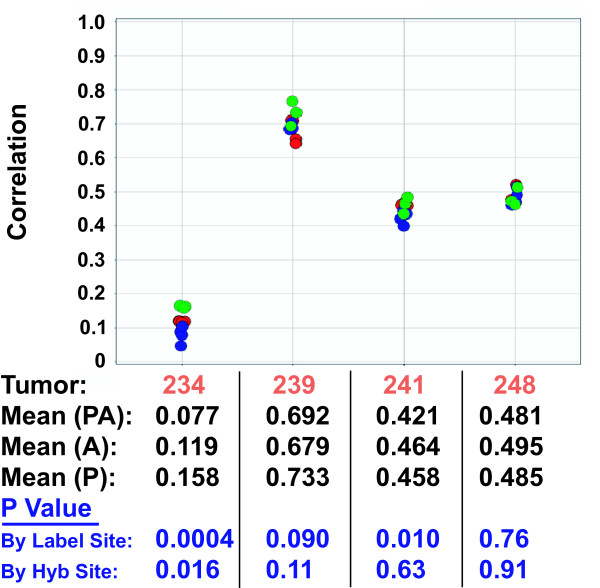
**70 gene signature correlation values between three laboratories**. 70-gene signature correlation values for the four tumors were determined for each hybridization done in three different laboratories. On the x-axis are the four different tumor samples, and on the y-axis are the correlation values for each dye-swapped pair. Green data points were labeled and hybridized in Paris, red data points were labeled in Amsterdam, and blue data points were labeled in California. The mean correlation values at each hybridization location, and the ANOVA P values when grouped by labeling and hybridization site are shown beneath the plot.

Figure 7 shows the 70-gene signature correlation values for each of the four tumors when labeled and hybridized in the Paris lab using different lots of arrays and reagents (green), and the results are compared with those obtained in California (red) and Amsterdam (blue). We performed an ANOVA analysis to determine whether the locations differed in the correlation value means for any of the tumors, as reflected by the ANOVA P values. We found that as in the comparison between just the Amsterdam and California sites, when grouped by labeling site, the correlation value distributions for tumors 234 and 241 were significantly different, while those for tumors 239 and 248 were not. When grouped by hybridization site, only tumor 234 was significantly different. Since the Paris samples were both labeled and hybridized in Paris, this probably reflects the very low P value of the labeling difference between sites. Thus, even at a third site, using different lots of reagents and arrays manufactured several months after the ones used by the first two labs, the 70-gene signature correlation values for each of the four tumors were very consistent.

## Discussion

In this study we examined the reproducibility of a 70-gene breast cancer signature in a series of experiments performed in three laboratories, one in Amsterdam, one in California, and one in Paris. In the first part of the study identical RNA samples were labeled and hybridized to identical microarrays using the same platform and protocols, in both the Amsterdam and California laboratories. Reproducibility of signals and ratios was measured for replicate assays in each laboratory. We found that the results were very reproducible between sites. The low noise across the entire platform, as shown by the reproducibility of replicate hybridizations (those done in the same laboratory with the same labeled material), allowed the averaging of the replicates, with the result that minor differences in the data became more apparent (Figure 5). In the second phase of the study, the same tumors were labeled and hybridized in the Paris laboratory. Despite being done several months later, and using different lots of microarrays and labeling reagents, the results from the third laboratory were in close agreement with those from the two other laboratories, giving another indication of the robustness of the measurement technology.

We took care to be sure the same operating protocols were used between all the laboratories, and the operators in all laboratories were well trained. We found that if variations in the wash protocol were introduced between laboratories, significant discrepancies in the results emerged (data not shown). It is clear from our findings and those of others [17] that microarray protocols must be uniform and strictly adhered to in order to achieve good reproducibility between laboratories and operators. However, as we show here, if this is done then reproducibility is very high.

A DNA microarray measurement can be considered as hundreds or thousands of simultaneous analytical measurements of the relative concentrations of mRNAs in a sample. In order to examine the analytical precision, accuracy, and detection limits of these measurements, several laboratories have published cross-platform and other comparisons of microarray measurements [17-19,23-29]. However, there has not been a detailed examination of the factors contributing to any observed variability in the measurements. A microarray measurement requires several distinct steps. The microarrays themselves must be printed, handled, and stored until use. The RNA sample is purified, labeled with fluorophores, possibly amplified, and possibly fragmented. The labeled sample is hybridized to the arrays, which are then washed, dried, and scanned. At each of these steps variation and errors can arise which could contribute to imprecision in the overall measurement. By using the same input RNAs, the same batches of arrays and reagents, and by exchanging labeled samples and hybridized slides between the Amsterdam and California laboratories, we were able to examine which steps exhibited the largest variation between the two sites.

It should be noted that the experimental setup used in this study cannot measure every possible source of variation. Since all of the hybridizations involving a common sample were hybridized to arrays on the same slide, and the replicate slides in each laboratory were hybridized on different days, we cannot determine whether any variation observed between the two replicate slides is due to slide-to-slide variability or day-to-day variability, or a combination of the two. However, since the experimental setup compounds both potential sources of variation, we would expect that any such differences would be maximized in this study. Despite this, the 70-gene signature correlation values did not vary significantly by hyb day (Figure 4).

Another possible source of variation is inter-individual variability. Since all the labelings and hybridizations done at each site were performed by single individuals, the cross-laboratory variability cannot be de-convoluted from the inter-individual variability. However, we would expect that if two different individuals took care to follow the exact protocols, as in this study, that interlaboratory variation would be greater than inter-individual variation, due to use of a different set of laboratory equipment (pipettes, hybridization ovens, etc.). Another study reported measuring the 70-gene signature correlation values of two tumor samples repeatedly in the same laboratory, by six different individuals, with very consistent results (14, and data not shown).

We found that the largest discrepancy between the Amsterdam and California sites was in the amplification/labeling step. This discrepancy was relatively small (about 0.02 in the log_10 _ratios, which amounts to a 5% difference in the actual expression ratio) but is detectable nonetheless. We used labeling kits from the same lots and purchased at the same time, so all labeling reagents were equivalent. While the labeling site differences were significant for only two of the four tumors when comparing the tumor signature correlation values, the differences extended to all four tumors when examining the log_10 _ratios of the 70 signature genes on an individual basis. This suggests that the differences seen on an individual gene level are relatively random, and cancel one another out when looking at the signature as a whole, which represents a correlation of the log_10 _ratios of all 70 genes and averages of measurements from three replicate features for each gene. The variation in individual genes did not correlate with the expression level of the genes, which differs from the findings of Dobbin et al. [19] who found that lower expressed genes were more variable between laboratories.

Several previous studies examined the cross-platform comparability of microarray measurements [17,18,20-26], with some studies reporting less variability between platforms than others. Our findings that array results on one platform performed with identical protocols are reproducible across laboratories are similar to the findings of other studies [17-21]. However, ours is the first report of the reproducibility of a gene expression signature comprised of a small, defined set of genes. Such signatures have great potential utility in biomedical research, toxicogenomics, pharmaceutical development, and diagnostics. Reproducibility across labs and over time is essential in all these application areas, and our results are an encouraging indication that microarray-based analysis of defined gene signature sets can yield highly robust and reproducible measurements.

## Conclusion

We tested the reproducibility of DNA microarray measurements by measuring a 70-gene breast cancer expression signature across three different laboratories. We found high intralaboratory and interlaboratory reproducibility when using strictly controlled standard operating procedures.

## Methods

### RNA samples

Total RNA from four breast tumors were isolated as previously described [1,2]. A pool of 105 breast tumor RNAs was prepared as a reference RNA, as described in Glas et al. [14]. 200 ng of total RNA from the breast tumor pool and the individual breast tumors were amplified and labeled with Cy3- and Cy5-CTP (PerkinElmer) using the T7-based Low RNA Input Fluorescent Linear Amplification Kit (Agilent Technologies, Santa Clara, CA). The same lot of labeling kit was used by both the California and Amsterdam laboratories, while a different lot was used by the Paris laboratory. Labeled RNAs were quantitated for yield and dye incorporation using a Nanodrop spectrophotometer. To ensure that equal amounts of RNA were hybridized in both labs, the RNA concentration for all samples was determined at one site.

### DNA microarrays

The DNA microarrays were fabricated by Agilent Technologies according to specifications provided by Agendia BV. The array design contained 1900 features of 60 mer oligonucleotide probes associated with the MammaPrint^® ^assay as previously designed and described by Glas et al. [14]. 232 features containing probes for 231 genes previously found to be associated with breast cancer outcome [1], plus ESR1 (estrogen receptor), were present in triplicate on the arrays (696 features total). 915 individual features containing probes for cellular genes were used for dye normalization between the Cy3 and Cy5 channels. The remaining 289 features contained various positive and negative control probes. The microarray slides contained 8 identical arrays per slide, which could each be individually hybridized [14].

### Microarray hybridization

Microarray hybridization was done according to the manufacturer's recommended protocol (Agilent Technologies). 200 ng each of Cy3- and Cy5-labeled RNA were hybridized to each array in a 45 ul total volume of hybridization buffer (Agilent Technologies) for 16 hours at 60C, followed by room temperature disassembly in 6× SSC/0.005% Triton X-102, a ten minute room-temperature wash in 1× SSC/0.005% Triton X-102, and a five minute room temperature wash in 0.1× SSC/0.005% Triton X-102. Slides were dried with filtered, compressed nitrogen and scanned immediately in a DNA Microarray Scanner (Agilent Technologies). After slides were scanned in the Amsterdam or California laboratories, they were then shipped overnight to the other laboratory for rescanning. Slides hybridized in Paris were not rescanned.

### Data analysis

Array images were extracted using Agilent Feature Extraction software, version A.7.5.1, per manufacturer's instructions. After subtraction of feature backgrounds the signals in the test and reference channels were normalized for consistency of the normalization features, as described in the Feature Extraction software documentation.

For the 232 genes with three replicate features per array, the signals for the triplicate features on each array were averaged [14]. For each breast tumor sample, the correlation coefficient of the level of expression of the 70 previously described breast cancer signature genes [1,2] with the previously determined average profile of these genes in tumors from a specific set of patients was calculated as previously described [1,2,14].

To assess reproducibility in this study, ANOVA P values were calculated using JMP 5.1 software (SAS). To determine the averaged log_10 _ratios of probes from the four arrays (two dye-swap pairs) that shared the same labeling and hybridization location, we took the probes for the 232 breast cancer-related genes [1,2] and eliminated all probes with signals of less than 15 counts, which is three times the additive background noise measured on the noisiest array.

## Authors' contributions

RAA participated in the study design, performed all the lab work in California, participated in the data analysis, and drafted the manuscript. AF participated in the study design, supervised and coordinated the lab work in Amsterdam, and helped draft the manuscript. BC participated in the study design, and did much of the data analysis. VL supervised and coordinated the lab work in Paris. AMG participated in the Mammaprint analysis. RP performed all the lab work in Amsterdam. AT participated in the data analysis. HR performed the data processing of the Paris data. FC aided in the protocol design. MSA was a study coordinator. LB supervised the work in California, and helped draft the manuscript. LJVV was the supervisor/project leader, and helped draft the manuscript. All authors read and approved the final manuscript.

## Supplementary Material

Additional file 1 **Person correlations of replicate hybridization pairs**. This Microsoft Excel file gives the Pearson correlation values of the sample and reference channels for the 8 pairs of replicate hybridizations for each of the four tumors. Click here for file
